# Organizational culture and sport: a narrative review and proposed synthesis framework

**DOI:** 10.3389/fspor.2026.1778505

**Published:** 2026-05-18

**Authors:** John Cairney, Stephen Townsend, Zoe Harrison, James Woodforde, Kai Wheeler, Samantha Mulcahy, Sjaan Gomersall

**Affiliations:** 1 School of Human Movement and Nutrition Sciences, University of Queensland, Brisbane, QLD, Australia; 2 Queensland Centre for Olympic and Paralympic Studies, University of Queensland, Brisbane, QLD, Australia; 3 Health and Well-Being Centre for Research Innovation, Brisbane, QLD, Australia

**Keywords:** community sport, elite sport, leadership, management, organizational culture, power, administration, theory

## Abstract

In September 2025, the Brisbane Lions won an improbable second consecutive Grand Final of the Australian Football League—the Australian equivalent of a National Football League (NFL) Superbowl Championship or the English Football Association (FA) Cup. With a relatively inexperienced team and haunted by almost two decades of mediocrity, the sporting press and public immediately began searching for the secret to their success. For the Lions, the reasons for their victory were neither a surprise nor a secret. In the immediate aftermath of the game, co-captain Harris Andrews stated: “We've set up a culture where guys can thrive.” The “culture” of a sporting organization is often cited as being key to building a successful sporting organization at both the elite and grassroots or recreational levels of competition. Despite the apparent centrality of this factor, definitions of what constitutes positive “culture” in sporting organizations remain elusive. Sporting organization culture is currently undertheorized and often understood only within the context of a specific body, club, or organization. This narrative review closes the theoretical gap and aims to provide a comprehensive analysis of the existing approaches to developing positive cultures within sporting organizations in Australian and internationally. To do so, we review six prominent models of organizational culture drawn from the broader literature in management and organizational studies. For each model, we examine whether and how it has been applied to sport organizations. We conclude by proposing an integrative framework that synthesizes insights from across these models to offer a new conceptual lens for understanding organizational culture in sport that can be adapted and applied to diverse settings.

## Introduction

In an era of überprofessional sport, there is an increasing recognition that the success of a sporting organization hinges not only upon the performance of its players and coaches, but also the efficacy of the “backroom” staff who oversee its administration. Whilst the importance of administrators and managers has generally been acknowledged by those within sport, it is a sign of their increasing prominence that the “culture” of sporting organizations is now part of a broader public discourse. In a recent example, “culture” was cited as a key reason for the Brisbane Lions winning a second consecutive (2024 & 2025) Grand Final in the Australian Football League. With a relatively inexperienced team and haunted by almost two decades of mediocrity, the sporting press and public began searching for the secret to their surprise success. In the immediate aftermath of the game, co-captain Harris Andrews stated: “We've set up a culture where guys can thrive.” Similar assertions are frequently made of other high profile sporting organizations, wherein their success or failure can be attributed to the effective functioning of the body's managers, administrators, lawyers, communications professionals, and other support staff. Despite the centrality of managerial and administrative functions in sporting organizations, definitions of what constitutes positive organizational “culture” in sport remain elusive. In this analysis, we survey the extant literature in fields relevant to organizational culture, to assess how the models and theories presented in these publications may be applied to sport, at elite and recreational levels of competition.

Organizational culture refers to the shared values, beliefs, assumptions, and norms that shape the social and psychological environment of an organization (1). It encompasses the symbolic systems, meanings, and everyday practices through which members interpret their experiences and navigate organizational life (2–4). Functioning as an implicit guide, culture influences how individuals behave, make decisions, communicate, and interact, shaping both strategic direction and day-to-day operations (5). Although intangible, culture is considered a critical determinant of organizational success, especially in relation to leadership (1, 6), performance (7, 8), change management (9, 10), and stakeholder engagement (11, 12).

The importance of organizational culture lies in its capacity to influence outcomes at multiple levels. Strong and aligned cultures can foster coherence, innovation, and adaptability (1, 7, 8), while fragmented or toxic cultures may contribute to dysfunction, resistance to change, and underperformance (3, 13, 14). Culture serves as a lens through which organizational members interpret events and actions, often reinforcing behaviors and practices over time (3). Understanding culture is therefore central to efforts to improve organizational effectiveness and resilience in both stable and turbulent contexts.

Studying organizational culture within the sport context is particularly compelling due to the unique characteristics and diversity of sport organizations. Sport comprises a broad ecosystem that includes national and international federations, governing bodies, professional and amateur teams, grassroots and community clubs, and hybrid commercial-social enterprises (15, 16). These entities operate in environments marked by high public scrutiny, intense competition, complex stakeholder relationships, strong emotional investments, and an obligation to operate in ways that reflect the public's high moral regard for sport. Cultural dynamics in sport can influence athlete wellbeing, governance integrity, diversity and inclusion efforts, and, as evidenced by the example provided earlier, even on-field performance (17).

Yet, despite its importance, organizational culture in sport remains under-theorized and often inconsistently applied in practice and research (18). Studies of organizational culture in sport tend to apply single theories, which are often informative but fail to account for the breadth and nuance of sporting organization structures, personnel, and mission. This paper will take a more integrated approach to understanding organizational culture in sport by combining key features from six models of organizational culture drawn from the broader literature in management and organizational studies.

The literature analyzed in this paper was selected by a phased narrative review process, which is outlined below. Compared with a systematic review, for example Bogale and Debela's (19) assessment of organizational culture theories, narrative review offers greater conceptual flexibility and is particularly suited to topics where the methods and evidence are heterogenous, and the boundaries of the field of enquiry are blurred. These are certainly features of organizational culture research, which melds qualitative and quantitative methodologies across multiple disciplines, including psychology, cultural studies, anthropology, sport management, business, and others.

This has resulted in a range of organizational culture theories, models, and frameworks which conceptualize “culture” in diverse ways, with a distinct schism between functionalist models (culture is a measurable product of an organization's operations) and interpretivist models (organizations are products of their cultures). Employing a narrative approach enabled us to target studies that employed models that could potentially straddle this theoretical divide. Through this narrative review, we selected models with both functionalist and interpretivist features, which could be dissected and reconstituted into a model that blends the functionalist preference for measurable outcomes with the interpretivist perspective of culture being mutable and shaped by shared assumptions, values, and meaning-making practices within the organization (1). As such, the publications included in this paper should not be viewed as an exhaustive survey of organizational culture literature, but rather a selection of articles that employed prominent models of organizational culture which suited these aims.

The process used to identify the six organizational culture models included in this synthesis occurred in several stages. First, we conducted an exploratory scoping exercise using ChatGPT to generate an initial list of influential and widely used theories and models of organizational culture. This step was intended only to identify candidate models and was not used as a basis for final inclusion decisions. Second, the models identified through this scoping exercise were cross-checked against recent narrative and systematic reviews of the organizational culture literature (19). This step ensured that the candidate models identified were consistently recognized in contemporary scholarship as foundational or widely applied frameworks for understanding organizational culture. Third, models were retained only if they satisfied the following criteria:

1. They explicitly conceptualized organizational culture as their primary phenomenon of interest.
2. They provided a framework for describing, classifying, or analyzing cultural patterns within organizations.
3. They were sufficiently generalizable to be applied across different organizational contexts rather than being tied to specific organizational outcomes or performance frameworks.

Applying these criteria resulted in the exclusion of several frameworks that were initially identified but did not meet the conceptual focus required for this synthesis. For example, the Denison model (7, 20) and the McKinsey 7S framework (21) were excluded because both embed culture within broader models of organizational performance and effectiveness. While these frameworks contain cultural elements, their primary purpose is strategic alignment and organizational performance analysis rather than the conceptualization of culture itself. Similarly, Goffee and Jones' Double S Cube model (6) was excluded because it defines organizational culture narrowly along only two dimensions (sociability and solidarity) providing a more limited typological perspective than the broader frameworks included in the present synthesis. The final set of six models therefore reflects frameworks that conceptualize culture as a fundamental organizational phenomenon rather than as a derivative component of strategy, structure, or performance.

For each model, we will examine whether and how it has been applied to the study of sport organizations, summarizing key findings and contributions. In the final section, we propose an integrative framework that synthesizes insights from across these models to offer a new conceptual lens for understanding organizational culture in sport. This framework aims to support understanding, guide the development of empirical research and practical interventions, and provide a foundation for analyzing cultural dynamics, while guiding purposeful cultural change within diverse sport settings.

## Schein's three-level framework of organizational culture

Edgar Schein's model of organizational culture is one of the most influential and enduring frameworks in the field of organizational studies. First articulated in *Organizational Culture and Leadership* (22) and refined in subsequent editions (1, 23, 24), the model conceptualizes culture as existing at three distinct but interrelated levels: artifacts (the visible and tangible elements of a culture, such as rituals, language, and physical layout), espoused values (the stated norms, philosophies, and beliefs that guide behavior), and basic underlying assumptions (the deeply held, often unconscious beliefs that truly shape organizational behavior). Schein (1) argues that understanding culture requires probing beyond surface-level manifestations to uncover the foundational assumptions that are taken for granted within an organization. This framework has served as the theoretical basis for numerous studies across sectors. In corporate change initiatives, Schein's model has been used to explain organizational transformations and leadership practices in large firms (2, 22). In healthcare systems, researchers have applied the model to assess and improve patient safety culture and interprofessional collaboration (25). In educational institutions, Schein's framework has informed studies of school leadership, academic culture, and teacher engagement (26, 27). Finally, in government and public administration, scholars have used Schein's model to explore how deeply rooted bureaucratic assumptions impact innovation and service delivery (28, 29). In each of these domains, the model has enabled researchers to diagnose cultural misalignments, explain resistance to change, and design effective transformation strategies grounded in a deeper understanding of how organizations function beneath the surface.

### Applications to sport contexts

Schein's model has also been applied to sport settings and has gained considerable traction as scholars seek to understand how cultural dynamics influence performance, identity, leadership, and organizational change. As Maitland, Hills, and Rhind (18) noted in their 2015 systematic review, the nascent body of literature on organizational culture in sport has accumulated in a seemingly *ad hoc* fashion, with studies being “thrown onto a pile” of research that lacks the coherence of more established topics. The authors did, however, note one clear trend in this body of research: the dominance of Schein's framework. More than half (18) of the 33 papers included in their review employed Schein's definition of “organizational culture”, indicating that this framework is foundational to understanding organizational culture in sport. One of the earliest sport-specific applications of Schein's framework was Scott's (30) conceptual article, which adapted Schein's notion of shared basic assumptions to the context of intercollegiate athletics. Scott emphasized the importance of cultural coherence in athletic departments and proposed that leaders could manage change more effectively by understanding the cultural layers within their organizations.

Empirical studies in sport have further expanded Schein's foundational model. MacIntosh and Doherty (31) examined a private fitness organization and compared leader intentions with employee perceptions, revealing gaps between espoused values and staff experiences. Their follow-up study (32) confirmed that such inconsistencies were associated with lower job satisfaction and increased turnover intentions. In the collegiate sport context, Schroeder (33) conducted interviews with successful NCAA coaches to explore how they established and modified team cultures. Framing his inquiry around Schein's model, Schroeder found that coaches manipulated artifacts (e.g., slogans, routines), articulated and reinforced espoused values, and recruited athletes who aligned with their preferred basic assumptions. Similarly, Schroeder (34) proposed a tailored application of Schein's model for athletic departments, incorporating elements such as institutional context and environmental influences. A comparable adaptation occurred in Frontiera's (35) study of culture change in professional sport, where team executives were found to shift cultural assumptions through deliberate strategies of value articulation, behavioral alignment, and embedding mechanisms, a process closely aligned with Schein's notions of “unfreezing” and “refreezing” assumptions.

More recent studies have confirmed the continued utility of Schein's model in sport settings. Jayakumar and Comeaux (36) investigated a Division I university athletic department and identified a disconnect between the institution's espoused commitment to academic achievement and its deeper assumptions that prioritized athletic performance. Bailey, Benson, and Bruner (37) explored the organizational culture of a CrossFit gym, finding that the gym's visible artifacts, shared values, and core assumptions were tightly aligned to create a strong and coherent culture. Norman, Rankin-Wright, and Allison (38) used Schein's model to examine gendered dynamics in the English Football Association, finding that while inclusion was a stated organizational value, underlying assumptions and everyday practices reinforced exclusionary norms. Junggren, Elbæk, and Stambulova (39) analyzed coaching practices in a Danish swimming club and found alignment across all three of Schein's cultural levels, with coaches' stated philosophies reflecting both their enacted practices and their underlying beliefs. Parent and MacIntosh (40) applied the model to the organizing committee for the 2010 Olympic Winter Games, showing how culture evolved under time pressure and how visible design choices and leadership mechanisms were used to socialize staff and instill common assumptions within a compressed time frame. Strittmatter and colleagues (41) draw on Schein's framework to examine young people's participation in decision-making in sport organizations. Their analysis shows that formal structures intended to support youth representation can be understood as cultural artifacts, while deeper assumptions about age, authority, and legitimacy continue to privilege adult decision-makers. The study demonstrates how underlying cultural assumptions can limit the effectiveness of formal participation mechanisms in sport governance (42). Further, Cole and Martin (42) also draw on Edgar Schein's three-level model of organizational culture to examine how culture can be intentionally developed and managed within sport teams. Using a case study of the Manawatu Turbos, the 2014 ITM Cup champions in New Zealand rugby, based on semi-structured interviews with coaches and team captains, the authors highlight the importance of formally recognizing culture as a core element of team management and emphasize how leadership practices and organizational structures can reinforce shared values, assumptions, and collective leadership within sport organizations.

Several consistent insights emerge from these studies. First, Schein's model is particularly useful in sport for diagnosing alignment or misalignment between what is said (espoused values) and what is done (artifacts and assumptions) (31–34, 36–38). Such misalignments have been shown to undermine leadership credibility, reduce morale, and inhibit efforts toward inclusion or athlete well-being. Second, Schein's concept of embedding mechanisms, leaders' actions, routines, and structural choices that transmit cultural meaning, has been validated in multiple sport contexts, especially in studies focusing on coaching and executive leadership (33, 35, 40). Finally, while Schein's model originated in the corporate world, its adaptability has enabled scholars to extend it to diverse sport settings, including elite teams, national governing bodies, and temporary organizations like Olympic committees. Schein's model may also be particularly appealing to scholars studying organizational culture in sport because two levels of the model—artifacts and espoused values—are explicit in sport, relative to other types of organization. Teams, clubs, and peak bodies display cultural artifacts such as songs, trophies, rituals, and mascots, and their values are frequently and loudly espoused by coaches, players, and administrators alike, particularly in professional and elite sport settings. Despite differences in scale and context, Schein's tripartite model offers a robust analytical lens for examining how shared meanings are constructed, maintained, and challenged in sport organizations.

## Competing values framework

The Competing Values Framework (CVF), developed by Robert Quinn and Kim Cameron in the 1980s, is a model for understanding and assessing organizational culture. It classifies culture into four archetypes—clan, adhocracy, market, and hierarchy—based on two axes of competing values: internal focus vs. external focus and flexibility vs. stability. The CVF has become one of the most widely used frameworks for analyzing organizational culture across disciplines, due to its versatility, clarity, and capacity to diagnose culture in relation to organizational performance and leadership. Each quadrant of the CVF reflects a distinct orientation: Clan cultures (internal focus and flexible) emphasize collaboration and employee development; Adhocracy cultures (external focus and flexible) prioritize innovation and adaptability; Market cultures (external focus and stable) focus on results, competition, and goal achievement; and Hierarchy cultures (internal focus and stable) value control, consistency, and stability (13).

Numerous peer-reviewed studies have validated the CVF in a range of contexts. For example, Hartnell, Ou, and Kinicki (8) conducted a meta-analysis of 84 empirical studies that assessed the CVF's different culture types against three outcome criteria: employee attitudes (wellbeing), operational effectiveness (efficiency), and financial effectiveness (performance). The review found a positive relationship between three of CVF's four different culture types and the aforementioned outcome criteria. Hierarchy cultures were omitted from the study results due to low statistical power. Hartnell and colleagues (8) concluded that clan cultures were most associated with employee wellbeing; market cultures with financial performance, and adhocracy, clan, and market cultures were all associated with efficiency. These results indicate that although the CVF's typologies have a positive correlation with most of the study's outcome measures, they should not be interpreted as entirely distinct or dichotomous in their effect. For example, adhocracy, clan, and market cultures were all positively aligned with organizational effectiveness, indicating that efficiently run organizations can, and should, employ these in concert with one another.

The CVF has also been applied in studies of public administration (43), healthcare organizations (44), and higher education (45), further establishing its relevance across sectors. The framework is often operationalized through the Organizational Culture Assessment Instrument (OCAI), which quantitatively measures an organization's cultural profile along the CVF dimensions.

### Applications to sport contexts

In the context of sport, the CVF has been widely used to analyze organizational culture in both professional and amateur environments, and in various national settings, including minor league baseball in the USA (46), Korean professional baseball (47), collegiate athletic departments in North America (48, 49), youth football clubs in Bulgaria (50), and amateur sport associations in Western Australia (51). These studies, and particularly (47) study of CVF in Korean professional baseball, indicate that the framework's structure and typology are robust across cultural boundaries. However, localized nuances in the expression of authority and collectivism suggest that while the four CVF types are broadly identifiable, their manifestation may vary in emphasis and practice.

A consistent finding across these studies and others, is that sport organizations often exhibit a blend of culture types, with clan and market cultures being the most prominent (46, 48, 50–52). For example, in American minor league baseball, organizations displayed strong clan and market characteristics, emphasizing both team cohesion and competitiveness (46). Similarly, Popkochev and Tsvetkov (50) found that youth football clubs in Bulgaria exhibited predominantly clan and hierarchy cultures, but expressed a desired shift toward market and adhocracy orientations.

Leadership-oriented studies have shown that transformational and democratic leadership styles are frequently linked to clan and adhocracy cultures, while authoritarian leadership aligns with hierarchy and market cultures (35, 48, 53). This trend has been identified in diverse settings, including college athletic departments (48, 49), European sports clubs (53), and professional teams in North America (35). These findings reinforce the notion that leadership style is both a reflection of and a contributor to cultural orientation, with participatory leadership fostering more collaborative and flexible cultural environments. Woolf et al. (49) used the Cultural Web to frame an educational case study of leadership change at a Canadian university athletics department. Woolf and colleagues tell the story of the new Dean using CVF-based questionnaire to understand his employees' experiences in the department, eventually revealing a troubling misalignment between the department's goal of athletic competitiveness and the stated values of college, which emphasized academic balance and participation. The case showed how the CVF can be effectively employed in real world settings to diagnose cultural problems.

Research has also supported the relationship between CVF-defined culture types and organizational performance in sport. Studies have shown that stronger, more clearly defined cultures, particularly those oriented toward clan or market values, are associated with higher performance, both in terms of on-field success and organizational outcomes (46, 50, 52). For instance, teams with strong market-clan cultures tend to have clearer values and expectations, which may facilitate better coordination and accountability (46, 50). These effects appear to be consistent across different levels of sport, from youth (50) to professional settings (35, 46), albeit from a limited body of literature. Similarly, from a governance perspective, Garmamo et al. (54) applied the CVF framework to analyze the tripartite relationship between “good” sport governance, organizational culture, and organizational structure in selected Ethiopian Olympic sport federations. The authors hypothesized that organizations that aligned clearly with one or more of the CVF culture types would see a direct impact on the governance of the organization—with quality of governance being measured by the Action for Good Governance in International Sports Organizations (AGGIS) tool. Instead, they found no direct relationship between CVF-culture-type and the quality of governance, but rather a mediated relationship wherein culture type shaped the organizational structure of the organization, and those organizations with clear structures tended to be governed better.

The use of CVF in sport is predominantly situated within a functionalist paradigm, where culture is viewed as something that can be measured, managed, and linked to performance outcomes. Scholars such as Wagstaff and Burton-Wylie (55) have called for broader engagement with interpretivist perspectives, arguing that subcultures, informal dynamics, and symbolic expressions of culture may be overlooked in highly quantitative approaches. Nonetheless, the widespread uptake of the CVF highlights the practical utility and conceptual accessibility of quantitative approaches for assessing organizational culture in sport.

## Handy's cultural typology

Charles Handy's cultural typology (56), which builds on Harrison's (57) earlier framework, offers one of the most enduring and accessible models for classifying organizational culture. Handy proposed four types—Power Culture, Role Culture, Task Culture, and Person Culture—each defined by differing configurations of power, authority, structure, and control. Drawing on metaphors from Greek mythology, Handy conceptualizes culture as a reflection of power distribution, task structure, and organizational values. Each type offers a distinctive lens for interpreting how organizations operate and how individuals relate to authority, responsibility, and each other.

Power cultures, symbolized by Zeus, are characterized by centralized authority and tight control. Decisions are made swiftly at the center, and influence is largely informal and based on personal connections rather than procedures. These cultures often thrive in entrepreneurial or autocratic environments where agility and charisma are prioritized. In contrast, Role cultures, associated with Apollo, are defined by hierarchy, bureaucracy, and clearly prescribed roles and responsibilities. Power is tied to position rather than personality, and such cultures are common in public sector agencies or large-scale organizations seeking consistency and stability (5, 56].

The third type, Task culture, symbolized by Athena, emerges in project-oriented or knowledge-intensive environments. In these organizations, influence is derived from expertise rather than formal authority, and success is measured by problem-solving and innovation. Task cultures often feature matrix structures and collaborative teams and are typically dynamic and adaptive (13). Lastly, Person cultures, metaphorically aligned with Dionysus, are relatively rare and occur in settings where the individual is considered more important than the organization. Common in professional partnerships such as law firms or consultancies, these cultures are non-hierarchical and prioritize autonomy and individual expression (56).

Handy's model has been widely used in organizational studies and management literature to interpret how decision-making authority is distributed, how work is coordinated, and how individuals relate to the organization. Handy's typology has been widely applied in organizational studies to explore leadership alignment (6), cultural barriers to change (58), sectoral differences in culture (60), and innovation dynamics across organizational types (59). Although more typological than deeply interpretive, Handy's categories provide an accessible entry point for diagnosing organizational form and decision-making logic, particularly in settings where formal and informal structures intersect.

### Applications to sport contexts

In sport, explicit applications of Handy's typology are less common than with frameworks such as Schein's levels of culture or the Competing Values Framework. However, the model has nonetheless informed sport management scholarship in both conceptual and empirical ways. A foundational example is Slack's (61) *Understanding Sport Organizations*, which introduced Handy's and Harrison's typologies to the sport context. Slack illustrated how small, informally governed clubs often function as Power cultures, dominated by a single figure (e.g., a founding president), while sport governing bodies may align more closely with Role cultures due to their bureaucratic structure and rule-bound procedures. Although Slack's analysis was conceptual and not empirical, it provided a foundation for later researchers to consider cultural types as a means of differentiating sport organizations.

A review by Jefferies, Coates, and Yi (62) further reinforced Handy's relevance, situating his typology among the major theoretical models that have shaped how scholars think about culture in sport. Their article emphasized that despite the dominance of Schein's model and the Competing Values Framework in applied research, Handy's four categories remain part of the conceptual backdrop. They observed that many studies in sport describe cultures in ways that implicitly align with Handy's types, such as highlighting hierarchical vs. collaborative dynamics, even if they do not use Handy's labels explicitly.

Among empirical studies of sporting organizations, Handy's typology has been used more as a heuristic reference point than as a central analytical tool. For example, Frontiera's (35) qualitative case study of professional sport teams in North America explored how leaders transformed team cultures by shifting decision-making power and reinforcing new values and practices. While the study heavily cites Schein's framework, the influence of Handy's typology is also present, and Frontiera's conclusions closely map onto his cultural types. Frontiera observed that many of the teams initially revolved around a dominant central figure, analogous to Handy's Power Culture or “Zeus” type. Through deliberate interventions, these organizations shifted toward more distributed, team-oriented cultures (resembling Handy's Task or “Athena” culture) where collaboration and initiative were emphasized.

Wagstaff and colleagues (63) also studied culture in elite association football (soccer) clubs in the UK, focusing on how relationships between stakeholders shaped organizational dynamics. Although their framework was not typological, the findings lend themselves to interpretation through Handy's lens. Clubs governed by authoritarian owners tended to exhibit characteristics of a Power culture, while those fostering player leadership and innovation displayed traits more consistent with a Task culture. These examples underscore the value of Handy's model in interpreting patterns of control, structure, and collaboration in professional sport organizations, even when it is not explicitly cited.

In university sport settings, Weese's (64) quantitative and qualitative study of campus recreation and athletic departments in the USA is often cited as a key early attempt to measure sport organization culture. Although he did not frame his study around Handy's four types, his classification of organizational environments as “bureaucratic,” “supportive,” and “innovative” echoes elements of Role, Person, and Task cultures, respectively. For example, departments characterized by formal structures and policies resembled Role cultures, whereas those emphasizing team innovation aligned with Task culture principles. Similarly, Colyer's (51) study of Western Australian sport organizations applied the Competing Values Framework, but the resulting culture types (e.g., clan, hierarchy) conceptually overlap with Handy's typology. Hierarchical cultures, for instance, reflect the same structural rigidity found in Handy's Role culture, while clan cultures share affinities with Person-oriented cultures focused on individual relationships and autonomy.

These studies collectively suggest that while Handy's cultural typology has not been the primary framework in most sport-specific research, its categories nonetheless resonate with observed cultural dynamics in many sport organizations (18, 61, 62). When drawing connections between empirical findings and Handy's model, it is essential to distinguish between studies that directly apply his typology and those that merely align with it conceptually (35, 51, 63). In doing so, researchers can maintain theoretical clarity while still leveraging Handy's insights to make sense of the diverse and sometimes conflicting cultural features that characterize sport organizations across different levels and contexts.

## Deal and Kennedy’s typology

Deal and Kennedy's (65) organizational culture model classifies organizational cultures along two central dimensions: the level of risk present in the organizational environment and the speed of feedback or reward following action. These dimensions yield four cultural archetypes: “tough-guy, macho” (high risk, quick feedback), “work-hard/play-hard” (low risk, quick feedback), “bet-your-company” (high risk, slow feedback), and “process” (low risk, slow feedback). In addition to this typology, Deal and Kennedy emphasized the importance of a strong, cohesive culture, what they termed a “thick” culture, defined by shared values, rituals, heroes, and beliefs. This model has been influential in corporate culture discourse and has also been adapted to analyze organizational dynamics in sport contexts.

Numerous peer-reviewed studies have applied Deal and Kennedy's framework in broader organizational contexts. For example, Parker (66) described how “bet-your-company” cultures characterize industries such as pharmaceuticals and aerospace, where high-risk decisions unfold over long timelines. The model has also been used to examine knowledge-sharing behavior in multinational corporations (67), where “process” cultures often exhibit resistance to innovation. Hatch and Schultz (11) drew on Deal and Kennedy's concepts of rituals and heroes to explain how organizations construct brand identity, while Ouchi and Wilkins (68) used the typology to explore cultural differentiation in public administration. These studies underscore the model's relevance across diverse sectors, from industrial and public services to global corporate environments.

### Applications to sport contexts

Two studies, in particular, offer clear illustrations of how Deal and Kennedy's typology and cultural elements framework can be used to understand sport organizations.

Armstrong (69) utilized Deal and Kennedy's cultural model to explore the notion of “cultural charisma” in professional sport organizations. Drawing on their emphasis on values, heroes, and rituals, the study conceptualized cultural charisma as a visible, embodied expression of organizational identity. Through a survey of 476 sport consumers in a large U.S. metropolitan area, the study found that perceived cultural charisma significantly predicted sport consumption behaviors. Deal and Kennedy's framework was used to interpret how a strong “work-hard/play-hard” culture, common in professional sports, could be leveraged as a branding asset.

Samur (70) conducted a qualitative interview-based study of professional sports clubs in Turkey that was explicitly framed through Cameron and Quinn's Competing Values Framework. The analysis identified four dominant cultural orientations—Solidarity and Cooperation, Innovative, Competitive, and Hierarchical—that closely aligned with the Clan, Adhocracy, Market, and Hierarchy cultures of the CVF. While the CVF provided the primary analytic lens, Samur also drew selectively on elements of Deal and Kennedy's cultural typology to interpret interviewee responses, particularly in characterizing rule-bound, bureaucratic club environments as akin to “process culture.” In this way, the study demonstrates that while existing models can be used in isolation to understand organizational culture in sport, it is also possible, and worthwhile, to selectively combine elements from different models to form a new, hybrid interpretive framework. In this instance, CVF-based analyses in sport may intersect conceptually with alternative culture typologies, supporting their interpretive relevance in a non-Western, club-based sport context. Later in this paper, we make our own attempt at designing a comprehensive hybrid model of organizational culture, and Samur's 2021 study is one example that shows a longer lineage of this practice within organizational culture research.

As is the case with Handy's cultural typology, Deal and Kennedy's model informs several studies of organizational culture in sport without being explicitly referenced. Many studies in sport leadership and organizational culture refer broadly to shared values or cultural strength, concepts emphasized by Deal and Kennedy, but few explicitly adopt the model's typology or terminology. For example, Weese (64, 71) and Kent & Weese (72), while influential in exploring the idea of strong cultures, do not explicitly cite Deal and Kennedy in their empirical frameworks. Similarly, case studies such as Cole and Martin (42), though referencing Deal and Kennedy as a foundational theory, primarily center other models like Schein's.

## Johnson and Scholes' cultural web

Johnson and Scholes' (73) Cultural Web framework is a tool for analyzing organizational culture by examining six interrelated elements: stories, symbols, rituals and routines, power structures, organizational structures, and control systems. These elements are arranged around a central organizational paradigm, which represents the set of core beliefs, assumptions, and values that are taken for granted within the organization and that inform all other cultural elements.

The six surrounding components function as both manifestations and reinforcers of this central paradigm. “Stories” refer to narratives shared within the organization that communicate its values and history; “symbols” encompass visual representations such as logos, dress codes, and office layout; “rituals and routines” involve habitual behaviors that indicate what is valued in practice; “power structures” reflect who holds influence and how it is exercised; “organizational structures” describe the formal and informal ways in which roles and responsibilities are organized; and “control systems” refer to the ways in which performance is measured and rewarded. Together, these elements provide a comprehensive map of the cultural landscape, enabling organizations to diagnose cultural strengths and weaknesses and to align cultural attributes with strategic objectives.

The model has been widely applied in management and organizational studies to explore how cultural elements influence strategic alignment (74) and organizational identity (11).

### Applications to sport contexts

In sport, the Cultural Web has been explicitly applied to uncover the gender dynamics of football governance (75), leadership development programs (76), talent development (77), and competitiveness (49). Peer-reviewed studies using this model span a range of sport settings and cultural contexts, from professional sport (75), to collegiate sport (49), and community-based organizations (76). These studies highlight how the Cultural Web enables researchers and practitioners to diagnose misalignments between cultural elements and organizational goals, thereby supporting targeted cultural change.

For example, de Haan and Norman (75) used the Cultural Web to explore how organizational culture influences men's engagement in gender equity efforts within European football associations. The analysis revealed that dominant stories, power structures, and rituals perpetuated male dominance and marginalization of women, even as formal equity initiatives were introduced. The authors argued that shifting these cultural elements, especially the paradigm around what constitutes success in football, was key to lasting change.

Edwards and Turnbull (76) integrated the Cultural Web into an evaluation of a leadership development initiative in a regional UK sports partnership. By examining changes across cultural elements over an 18-month period, they demonstrated how structural routines and power dynamics could obstruct leadership application. The framework provided a basis for identifying and addressing cultural barriers to program impact.

English (77) applied the Cultural Web to examine South Africa's cricket talent development system. This PhD thesis used interviews with coaches, administrators, and athletes, and study found that entrenched power structures and fragmented subcultures impeded cohesive player development. The framework illuminated how organizational structures and values were misaligned with post-apartheid ideals, making a case for cultural realignment.

The extant literature shows that Johnson and Schole's cultural web is not only well suited to understanding the internal dynamics of sporting organizations but also how these bodies respond to broader cultural imperatives, as illustrated by the failed efforts of cricket authorities to adapt to changed racial politics in post-apartheid South Africa (77), or gender equity in the case of European football (75). The integration of this model is particularly important because its interpretivist approach to culture balances the more functionalist tendencies of foundational models such as Schein and Hofstede.

## Hofstede's cultural dimensions

Geert Hofstede's cultural dimensions theory is among the most widely cited frameworks in cross-cultural management and international organizational research. Based on large-scale surveys of IBM employees across multiple countries in the 1970s and extended in subsequent decades, the model initially identified four dimensions of national culture—Power Distance, Uncertainty Avoidance, Individualism vs. Collectivism, and Masculinity vs. Femininity—later expanded to six with the addition of Long-Term Orientation and Indulgence vs. Restraint (78–80). The model conceptualizes culture as shared values at the national level that influence how individuals think, behave, and relate to institutions. These dimensions have been extensively applied across different cultural settings to understand leadership preferences (6), organizational design (81), decision-making processes (82), communication patterns (83), and employee motivation (84, 85).

### Applications to sport contexts

Hofstede's framework has been adapted to investigate how national cultural values shape athlete behavior, coach–athlete dynamics, team performance, fan engagement, and organizational practices. One line of inquiry has explored the effect of cultural distance on international labor mobility in elite sport. Jarjabka, Fűrész, and Havran (86), for instance, analyzed over 34,000 football player transfers and found that athletes were significantly less likely to migrate between countries with large differences on Hofstede's dimensions. Cultural distance, measured using the Kogut and Singh (87) index derived from Hofstede's scores, was shown to reduce player movement even after controlling for economic incentives. In a complementary study, Bosker and Gürtler (88) used a longitudinal performance model to assess how cultural differences affected individual soccer players after transferring between countries. Interestingly, while initial adaptation to a culturally distant environment was difficult, players who successfully integrated into new cultural contexts often outperform peers, suggesting long-term gains in performance. These studies demonstrate how Hofstede's framework can help explain both the barriers and benefits of cross-cultural transitions in professional sport.

Other researchers have extended Hofstede's model to examine grassroots and recreational sport behaviors. Suominen (89) conducted a cross-national study using data from 34 countries to examine how national culture relates to sport spectating (“fanatic participation”) and physical activity (“energetic participation”). The analysis found that people from individualistic and low–uncertainty-avoidance cultures were more likely to engage in both spectating and participation, while those from more collectivist or uncertainty-averse societies showed lower engagement. Cultural indulgence (vs. restraint) was also associated with greater female participation, highlighting how Hofstede's values shape even everyday sport behaviors at the population level.

Hofstede's dimensions have also been used to segment sport tourists. In a study of international participants at the 2005 Gold Coast Airport Marathon, Funk and Bruun (90) found that cultural background influenced motivation to travel and compete. Using Hofstede's cultural clusters, they demonstrated that runners from different regions (e.g., East Asia vs. Anglo countries) varied in the importance they placed on experiential and educational aspects of the event. This work illustrates the model's utility in sport marketing and event management, particularly in understanding how culturally grounded values influence consumer behavior.

In team contexts, Razali, Radzi, and Husin (91) explored how differences in coaches' cultural backgrounds (local vs. expatriate) moderated the relationship between leadership style and athlete satisfaction in Malaysia's high-performance sport system. Drawing on Hofstede's national profiles, such as Malaysia's high “Power Distance”, the authors interpreted why athletes responded differently to directive or democratic leadership styles depending on the coach's origin. Athletes often preferred local coaches whose leadership behaviors aligned more closely with prevailing cultural expectations. This study applied Hofstede's framework to better understand intercultural coaching effectiveness. Maderer, Holtbrügge, and Schuster (92) examined European professional football squads and found that greater cultural distance among players, calculated using Hofstede's individualism/collectivism measure, was associated with poorer team performance, likely due to communication barriers and subgroup dynamics.

At the organizational level, Hofstede's framework has been used to analyze governance and structural outcomes. Ahn and Cunningham (93) investigated gender equity on National Olympic Committee boards across 125 countries and found that lower Power Distance, lower Masculinity, and lower Uncertainty Avoidance scores predicted higher levels of women's representation. In another global-level study, Sava, Joldz, and Olar (94) linked national sport performance to cultural values, showing that whilst economic factors (represented as national GDP) are important, cultural factors such as Hofstede's measures of individualism vs. collectivism, Long-Term Orientation, and a strong Achievement orientation (related to Masculinity) were also highly influential in predicting Olympic success.

These studies illustrate the breadth of Hofstede's application across sport research domains, from elite performance to grassroots participation, and from athlete migration to sport governance. While the model has been criticized for its deterministic assumptions and static national categories (95, 96), its clarity and scalability make it a powerful tool for comparative sport research (97). Importantly, researchers continue to adapt Hofstede's framework to address both individual-level dynamics, such as athlete adaptation and satisfaction (88, 91), and systemic issues such as governance diversity and national sporting performance (93, 94). This ongoing evolution reaffirms the model's relevance across levels of analysis and across diverse areas of sport.

## Organizational culture synthesis framework

**Figure 1:**
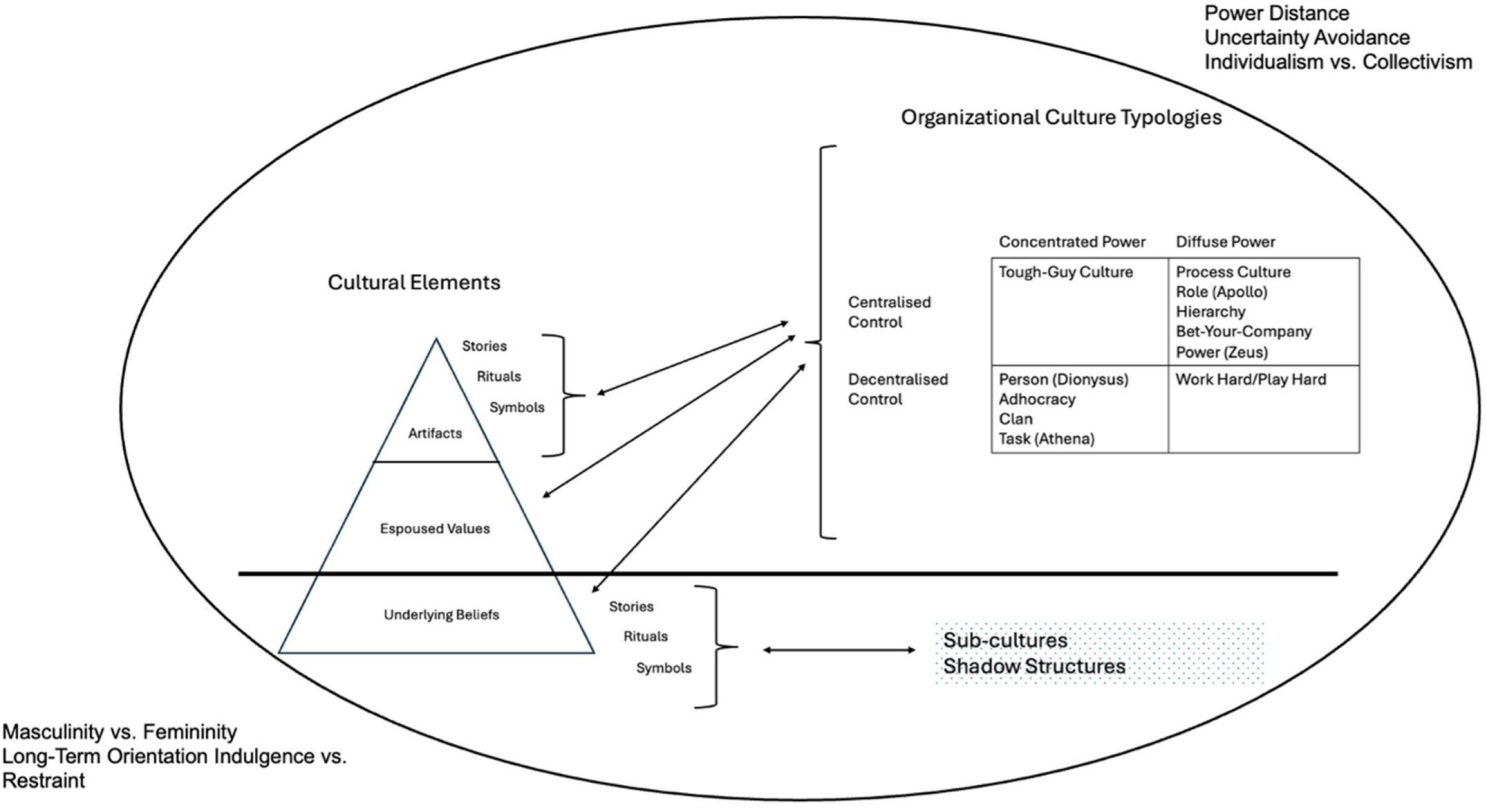
The integrative model of organizational culture in sport, based upon the key features of the six orgz.

The integrative framework developed here (Figure 1) brings together the six foundational models of organizational culture described in this review to represent the layered, expressive, typological, and contextual dimensions of culture within organizations. The purpose of this synthesis is to highlight areas of overlap and complementarity among these models while preserving their unique conceptual contributions, ultimately producing a fuller, richer account of how culture operates within organizational systems. At its core is Schein's (1) three-level model, which distinguishes between surface-level artifacts, articulated values, and the deeper, often unconscious, assumptions that guide behavior. Culture is thus understood as a multi-level phenomenon, what is visible or declared may not align with what is truly believed or enacted. Building on this foundation, the framework incorporates the expressive dimensions of culture highlighted in both Deal and Kennedy's (65) typology and Johnson and Scholes' (73) Cultural Web. These models identify stories (including heroes), rituals, and symbols as central mechanisms through which values are conveyed and culture is reinforced over time. While these elements operate visibly, they also serve to embed and sustain deeper assumptions, linking observable behavior with organizational meaning systems.

The model in Figure 1 introduces a horizontal plane that represents two distinct but complementary forms of cultural depth below the surface. The first refers to the unconscious cognitive domain, captured in Schein's (1) notion of basic underlying assumptions. These are deeply ingrained beliefs about time, reality, human nature, and relationships that are so taken-for-granted they are rarely questioned. They form the foundation for perception and action, functioning as the mental infrastructure of organizational life. These assumptions are not inherently problematic; in many cases, they provide stability, coherence, and shared meaning. However, they can become sites of tension, particularly when they conflict with newer espoused values or formal initiatives, such as when an organization promotes teamwork while operating on an unspoken belief in individual achievement and internal competition. This kind of misalignment can create resistance, inconsistency, or incoherence across cultural layers. The diagnostic value of this insight has been demonstrated in various studies, including in sport, where researchers have used Schein's framework to identify how conflicts between stated values and embedded assumptions can undermine leadership, morale, and change efforts (31–34, 36–38).

The second below-the-surface dimension represents the informal social domain of organizational life, what might be termed the “underground” or relational structures of culture. This includes subcultures (3, 98, 99) and shadow structures (100), the informal networks, routines, and practices that are not formally recognized by the organization but can significantly shape communication, influence, and action. These structures may support, bypass, or resist the dominant culture, and often arise in response to ambiguity, constraint, or lived experience within the organization. While they are not visible in official charts or documents, they play a crucial role in how people interpret and navigate their environment.

Importantly, stories, rituals, and symbols that operate below the surface are not best understood as arising from basic assumptions alone. Instead, they are often generated by and reinforce subcultures and shadow structures. For example, informal stories that circulate among mid-level staff about a leader's inconsistencies may reinforce a shared subcultural identity that values autonomy or skepticism. Likewise, recurring informal rituals (e.g., end-of-project celebrations that exclude certain groups within the organization) may express latent boundaries or contested hierarchies. These cultural elements are reciprocally linked to informal social formations: they give symbolic form to underground practices and simultaneously reproduce the values and norms embedded in those practices (59, 101).

To reflect the distinction and dynamic interaction between these two domains, the model includes a dotted box connecting subcultures and shadow structures to the stories, rituals, and symbols they generate and sustain. This dotted box indicates that these informal cultural processes are contingent, fluid, and often improvisational, being shaped by context and power dynamics rather than formal design. While the reciprocal influence between surface-level culture and structural forms is represented by solid arrows, these dotted connections highlight the unofficial and evolving nature of informal cultural life and its potential to either stabilize or challenge dominant norms.

In addition to its layered and typological architecture, the model incorporates double-headed arrows to indicate the reciprocal influence between cultural elements and structural forms, that is, the cultural typologies such as Adhocracy, Hierarchy, or Power culture. This two-way dynamic occurs both above and below the surface. Above the surface, formal symbols, rituals, and stories (e.g., leadership awards, branding narratives, or onboarding ceremonies) often reflect the dominant cultural form, such as a Clan culture's emphasis on family metaphors and peer recognition. Yet once institutionalized, these elements also stabilize and reinforce the form itself (2, 13). For example, in a Hierarchy culture, repeated references to “chain of command” in stories or symbols may deepen shared expectations for bureaucratic order, making the culture more self-reinforcing. Below the surface, informal stories, rituals, and symbols, those not formally sanctioned by the organization, circulate within subcultures or shadow structures and can either align with or resist the dominant form (3, 59, 99). In an Adhocracy culture, for instance, informal rituals of spontaneous idea-sharing may support the broader norm of innovation. Conversely, in a Power culture (56), underground humor or storytelling that mocks leadership could quietly undermine authority while reinforcing an oppositional subculture. These cultural elements both shape and are shaped by the structural logics of the organization, particularly how power is distributed and how control is exercised. Notably, this reciprocity is strongest at the levels of artifacts and espoused values, while basic assumptions tend to resist change, often shifting only through sustained dissonance, internal contradiction, or deliberate leadership intervention (1). The arrows in the model thus reflect the generative feedback loops that animate cultural life, how everyday expressions of meaning help to construct, entrench, or erode the very structural forms within which they operate.

The framework also brings together a range of typologies from the literature, which have long been used to classify and compare different kinds of organizational cultures. These typologies—drawn from Handy (56), Cameron and Quinn (13), and Deal and Kennedy (65)—are here unified within a 2 × 2 matrix organized by the dimensions of power and control. Power is defined as the extent to which authority and influence are either concentrated in a centralized figure or shared across the organization (56, 101), while control refers to the degree of formalization and regulation within organizational systems, ranging from decentralized and flexible to centralized and tightly structured [Ouchi (103); (13)]. The resulting typological map (summarized in Table 1) clusters culture types into quadrants based on how they distribute authority and manage behavior. Our decision to focus on these two dimensions reflects their central role in organizational theory as fundamental mechanisms through which authority is distributed and behavior is coordinated within organizations [(101); Ouchi (103)]. By abstracting across the six frameworks included in our analysis, we develop a parsimonious integrative model that enables the classification and comparison of different organizational cultures while preserving the core theoretical insights of the original typologies. For example, the top-right quadrant (concentrated power, centralized control) includes Role cultures (56), Hierarchy cultures (13), and Process cultures (65), each of which emphasize predictability, accountability, and bureaucratic order. The top-left quadrant (diffuse power, centralized control) includes Task cultures (56), Clan cultures (13), and Work-Hard/Play-Hard cultures (65), which prioritize performance and collaboration within a structured environment. The bottom-left quadrant (diffuse power, decentralized control) contains Person cultures (56) and Adhocracy cultures (13), which value autonomy, creativity, and emergent leadership. Finally, the bottom-right quadrant (concentrated power, decentralized control) includes Power cultures (56), Tough-Guy cultures (65), and Market cultures (13), which feature strong individual leadership and outcome-driven performance in loosely regulated systems. This matrix enables comparative insights across cultural types while highlighting the structural logics that underpin each one (5, 13, 56, 65).

**Table 1:** Twelve cultural typologies, arranged in ascending order of leadership power and control.

Typology	Theorist(s)	Brief Description	Quadrant Location	Justification
Adhocracy	Cameron & Quinn (12)	Dynamic, innovative, and entrepreneurial; values creativity and risk-taking.	Low Power/Low Control	Power distributed to innovators; minimal control to allow for agility and risk-taking.
Person (Dionysus)	Handy (56)	Individual-focused; exists to serve members, highly autonomous.	Low Power/Low Control	Power is diffuse, serving individuals; control mechanisms are nearly absent.
Clan	Cameron & Quinn (12)	Friendly, family-like; emphasizes teamwork, cohesion, and participation.	Low Power/Moderate Control	Decentralized, with shared norms guiding behavior; moderate coordination via trust.
Task (Athena)	Handy (56)	Project-based, expertise-driven; flexible and pragmatic problem-solving.	Low Power/Moderate Control	Power shifts based on expertise; guided by flexible, task-based control.
Work-Hard/Play-Hard	Deal & Kennedy (64)	Customer-focused, energetic, performance-driven with peer rewards.	Moderate Power/Moderate Control	Power is partially centralized but balanced with peer-based motivation; moderate control.
Tough-Guy Culture	Deal & Kennedy (64)	High risk, immediate feedback; individual stars dominate the culture.	High Power/Low Control	High-pressure, high-risk with informal power around star performers; limited formal control.
Hierarchy	Cameron & Quinn (12)	Structured and rule-bound; values stability, control, and efficiency.	High Power/High Control	Top-down authority and formal procedures dominate; clear hierarchy and tight rules.
Process Culture	Deal & Kennedy (64)	Low-risk, slow-feedback; focused on process, precision, and bureaucracy.	High Power/High Control	Extensive process adherence with senior control; formal structure dictates behavior.
Role (Apollo)	Handy (56)	Bureaucratic and role-oriented; stability through rules and structures.	High Power/High Control	Structured roles with concentrated authority and standardized behavior expectations.
Power (Zeus)	Handy (56)	Charismatic, centralized authority; power rests in strong individual leaders.	High Power/High Control	Leader-centric authority with strong top-down influence; behavioral control through loyalty.
Market	Cameron & Quinn (12)	Competitive, goal-focused; values outcomes, efficiency, and performance.	High Power/High Control	Performance-oriented with top-down goals and metrics enforcing control.
Bet-Your-Company	Deal & Kennedy (64)	High stakes with delayed results; emphasis on planning and long-term bets.	High Power/High Control	Strategic decision-making from the top; structured planning leads to tight control.

Finally, the outermost layer of the model acknowledges the role of national cultures or mores as conceptualized in Hofstede's (79) framework. National-level values create the societal conditions in which organizational cultures are formed, interpreted, and sustained. These macro-level influences do not determine internal cultures but provide a cultural envelope that shapes what is possible, acceptable, or expected within an organizational setting (97). For example, in high power-distance societies, hierarchical organizational structures may be more culturally congruent than in more egalitarian settings. By situating national culture at the outer boundary, the model reflects how organizational life is nested within broader societal logics, adding an essential layer of context to any analysis of cultural dynamics. Whilst some of the cultural distinctions described in Hofstede's model (for example “masculinity” vs. “femininity” and “individualism” vs. “collectivism”) may be more blurred for 21st century sporting organizations, the model is incorporated into this new synthesis because we believe that national and cultural mores are important to understanding how sporting organizations function. This is particularly true of peak bodies and professional or representative sporting organizations, which often have a multicultural membership and are simultaneously expected to reflect the imagined values of their nation, region, or people.

## A multi-level perspective on sport culture

**Figure 2:**
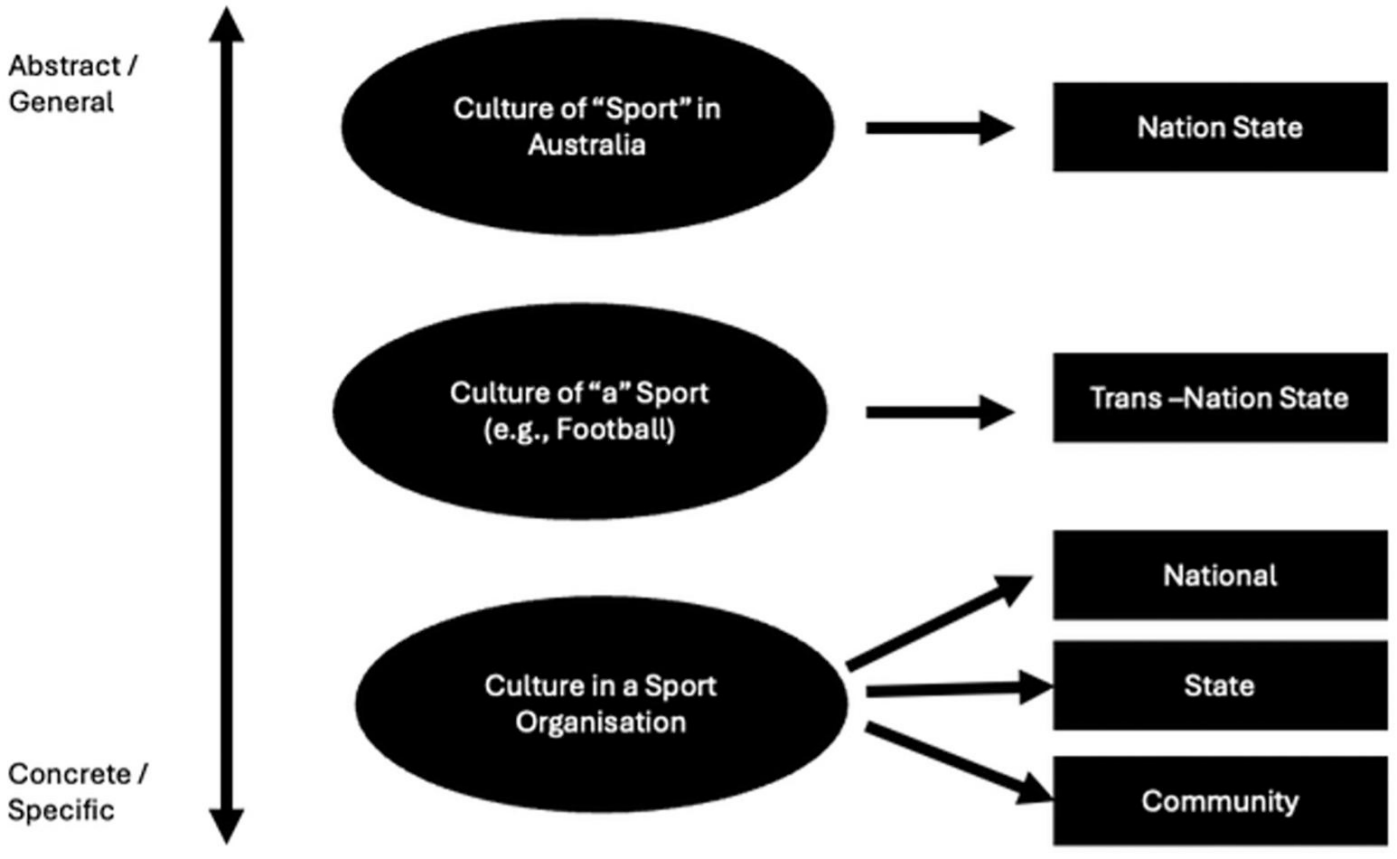
Multi-level model of sport culture.

What also emerged from our narrative review was that sport possesses unique cultural features that are not fully captured by traditional models of organizational culture, which are largely derived from business or general management contexts. These models often overlook the multi-layered and intersecting cultural identities embedded within sport. To address this gap, our team developed a complementary model to better represent the layered nature of sport culture. This Multi-Level Model of Sport Culture reflects how sport culture operates simultaneously at multiple levels of abstraction and specificity (see Figure 2).

At the most abstract level is the culture of “Sport” within a nation. This refers to how sport, as a general institution, is understood, valued, and practiced as part of a country's identity. Australia is used as the example in Figure 2 for the sake of rhetorical clarity, but any nation, region, or culture could be substituted here. In Australia, Australian rules football, as played in the elite “AFL” (Australian Football League) and AFLW (Australian Football League for Women) is deeply embedded in national culture, just as ice hockey is in Canada or baseball in the United States. This level of culture is shaped by broader national narratives, public policies, symbolic rituals, and the collective celebration or villainization of prominent sportspeople, that tie sport to civic identity and collective memory.

The second level is the culture of a specific sport, such as football (soccer), diving, or athletics. These sport-specific cultures often transcend national boundaries and reflect transnational norms, values, and identities. For instance, global football culture is defined by shared understandings of fair play, rivalry, and passion, yet it also allows for distinct local expressions, such as the stylistic differences between French and Brazilian football. This level highlights the hybrid and fluid nature of sport culture across geographic contexts.

At the most concrete level is the culture within a sport organization. This encompasses the shared beliefs, behaviors, and structural norms that shape how clubs, federations, and leagues operate. Organizational culture in sport functions at multiple scales, including national governing bodies, state or provincial associations, and grassroots community clubs. These localized cultures may contain subcultures and are often shaped by contextual factors such as demographics, funding models, and patterns of participation.

This multi-level model helps us understand that sport culture is simultaneously shaped by national identity and political structures, shared across global sport communities, and expressed in the daily practices of specific sport organizations. By conceptualizing culture in this layered and interconnected way, the model provides a powerful framework for analyzing alignment, influence, and tension across the sport system.

## Potential applications in sport

This integrative model holds particular value in the context of sport, where organizational cultures are often deeply embedded, multi-layered, and shaped by competing logics, such as high performance, community engagement, and athlete well-being. Sport organizations operate within complex ecosystems where governance structures, performance pressures, media narratives, and stakeholder expectations intersect with internal values and routines. The model allows for a detailed analysis of these dynamics by enabling practitioners and researchers to identify how cultural artifacts, such as team rituals, coaching language, or performance review formats, align or clash with espoused values like inclusivity or athlete development. More critically, it provides a lens through which to assess the underlying assumptions that may remain hidden yet drive behaviors, such as beliefs about toughness, sacrifice, or hierarchy in leadership. These assumptions often persist even when formal values have shifted, making cultural change efforts in sport particularly challenging.

For example, a high-performance national sporting body may articulate values of athlete welfare and holistic development, while simultaneously maintaining reward systems and leadership structures that reinforce a win-at-all-costs mentality. Applying the model, analysts could identify a misalignment between the espoused values and the organization's typology, likely situated in the high-control, concentrated-power quadrant, consistent with a Market or Bet-Your-Company culture type.

By drawing on the depth of Schein's (1) framework and the typological mapping of Deal and Kennedy (65), Handy (56), and Cameron and Quinn (13), this new synthesized model helps surface tensions between what the organization says it values and what its systems actually reinforce. This has implications not only for performance and ethics but also for trust, retention, and the long-term sustainability of sport cultures. The proposed synthesized model can be applied by leaders and administrators in sporting organizations, particularly during times of change or transition, such as moving to a new regulatory or policy structure, responding to scandal or competitive failure, or adapting to new technologies. In this way, the model serves as both a diagnostic and developmental tool for sport leaders seeking to build more coherent, aligned, and adaptive organizational cultures, and could be integrated into sports management courses at higher education institutions and short course offerings.

Future research may test the applicability of this new model by using it to analyze a range of sporting organizations, particularly organizations whose reasons for success or failure may initially seem complex or opaque. Along these lines, it may be fruitful to examine how different forms of organizational culture promote or inhibit inclusivity and equity in sport. This is particularly relevant to 21st century sporting organizations whose performance is often judged not only on profit or on-field performance, but also the diversity of its members and how equitably it distributes opportunities for participation. Acknowledging this, future studies of organizational culture in sport may seek to augment the proposed model with more interpretivist approaches. We acknowledge that our synthesized model reflects the functionalist tendencies of existing research into organizational culture and sport management, because these fields are so often concerned with measurable performance. Whilst this study and the resulting synthesized framework do integrate interpretivist elements, particularly taken from Johnson and Scholes' (73) Cultural Web theory, the functionalist leanings of sport management research are present in this new model.

In an era of increasing sociocultural ambiguity in sport—whether that be related to participant demographics, politics, social license, and a range of other issues—interpretivist approaches that see culture as constructed and therefore subject to constant re-negotiation may become central. For example, future research may look to incorporate features of interpretivist models, as McDougall and colleagues (102) did in their investigation of the Martin and Meyerson Three Perspective Approach, which explicitly accounts for contested, ambiguous, and marginalized expressions of culture within organizations. We therefore encourage future researchers to critique and adapt this model to further incorporate interpretivist views of organizational culture, where appropriate. Modern sporting organizations are faced with a myriad of challenges and opportunities related to equity and inclusion, such as the integration of transgender, intersex and gender diverse athletes, or the growing numbers of women participating in traditionally masculine high-contact sports such as football or boxing. It is likely that some forms of organizational culture will be better able to adapt to these changing athlete populations, however, it would be premature here to hypothesize which are likely to be more successful at this. For example, it may seem logical that organizations with diffused power and decentralized control will be better able to adjust their cultural elements to incorporate new and diverse participants, but it is also foreseeable that an organization with a powerful and progressive leader, who is focused on inclusion, may be able to steer their organization toward these outcomes.
